# Can we link foot ulcer with risk factors in diabetics? A study in a tertiary care hospital

**DOI:** 10.12669/pjms.346.16199

**Published:** 2018

**Authors:** Muhammad Imran Hassan Khan, Usama Azhar, Fizza Zubair, Zohaib Abbas Khan

**Affiliations:** 1 *Dr. M. Imran Hasan Khan, MBBS (Pb) MRCP (UK), FCPS (MED), MRCPS (Glas), FRCP (Edin) FRCP (Glas) FRCP (LONDON) Department of Medicine, Ameer-ud-Din Medical College/ PGMI, Lahore General Hospital, Lahore, Pakistan*; 2 *Usama Azhar, (Final Year MBBS) Department of Medicine, Ameer-ud-Din Medical College/ PGMI, Lahore General Hospital, Lahore, Pakistan*; 3 *Fizza Zubair, (Final Year MBBS) Department of Medicine, Ameer-ud-Din Medical College/ PGMI, Lahore General Hospital, Lahore, Pakistan*; 4 *Zohaib Abbas Khan, Director technical (Add), Drug Testing Laboratory, Punjab, Rawalpindi*

**Keywords:** Diabetic Foot Ulceration, Link, Neuropathy, Retinopathy, Medication

## Abstract

**Objective:**

Although many studies worldwide explained the risk factors for developing Diabetic Foot Ulceration (DFU), little has been done to assess medical factors in DFU formation and link them in patients of Pakistan. This study aimed to link the DFU with different risk factors.

**Methods:**

This descriptive cross-sectional retrospective study was conducted in Diabetes Endocrine and Metabolic Centre / Post Graduate Medical Institute / Lahore General Hospital. Data of all patients presenting between July 2017 to June 2018 were analyzed for risk factors. Analysis was done on SPSS version 21.

**Results:**

Total of 3301 patient were seen during this period, out of which 2052 patient data was picked up as it was complete in respect to the information needed. Middle age, Male gender, Type 2 diabetes, and Hypertension, were insignificantly co-related. High waist circumference, Comorbidity like Neuropathy, Dyslipidemia, Greater body mass index, Poor compliance with Medication and type of medication used (combination of oral and injectable) were found statistically significant predictor for DFU. However retinopathy was not found to be a risk factor of DFU. This result was statistically significant.

**Conclusion:**

Factors like obesity, waist circumference, combination of oral along with injectable therapies, neuropathy, dyslipidemia, retinopathy and poor compliance with medication were statistically significant and can be strongly linked with diabetic foot ulcer. Middle age, Male gender, Type 2 diabetes, and Hypertension were insignificantly co-related. However, further studies are needed in larger population to support these findings.

## INTRODUCTION

The increasing incidence of foot ulcer in patient with diabetes mellitus has placed a huge burden on health care system.^1^ Among 347 million people suffering from diabetes worldwide,^2^ it was estimated that one in 20 of these patients will develop a foot ulcer in one year, and over 10% of these ulcers will end up in an amputation.^3^ Hence, they contributed to societal cost of diabetes as foot problems in diabetic patients were the commonest cause of admission to hospital with risk of amputation estimated to increase 15–20 times. In fact, some 50% of all lower limb amputations were done in diabetic patients.^4^ In addition to increased cost of treating foot ulcer patient especially those requiring inpatient care, limb amputation was a major impact on the individual. It not only distorted body image, but also caused increased dependency and loss of productivity with patients reporting stigma, social isolation, loss of social role, and unemployment.^5^

In order to prevent this morbidity, many studies were conducted globally by investigators to find out the risk factors contributing to occurrence and recurrence of foot ulcers in diabetic patients.^6^ However, there was lack of sufficient research data available which could specifically highlight the incidence of diabetic foot ulcers in relation to different risk factors, including medication being used by the patient. It was part of a study done in Iraq which showed that patients using a combination of insulin and oral antidiabetic agents were more prone to develop diabetic foot ulcer.^6^ The same finding was observed in an Indian study which showed that usage of a combination of insulin plus oral hypoglycemic agents to be the most important risk factor.^7^ It was observed that combination therapy was commonly given to diabetic patients probably because it was a progressive disease that became less responsive to treatment with time.^8^ Moreover, use of many medications decreased patient compliance to therapy,^9^ which further meant loss of glycemic control^10^ and increasing DFU risk to the patient.^11^

Although many studies worldwide have explained the risk factors for developing DFU, little has been published to assess the involvement of clinical factors in Diabetic Foot Ulcer (DFU) among the patients of Pakistan. This study aimed to link diabetic foot ulcer with different risk factors in our population. In addition to this we also saw the effect of these factors contributing in DFU formation. In this way we hoped to contribute in prevention of disastrous consequence of diabetic foot ulcer.

## METHODS

The present cross-sectional study was carried out in an outpatient setting of Diabetes Endocrine and Metabolic center of Lahore General Hospital. It included all diabetes mellitus patients who attended the clinic between July 2017 and June 2018, collected on Microsoft excel sheet. The study was approved by the Ethical Review Committee of Ameer-ud-Din-Medical College/Post Graduate Medical Institute/Lahore General Hospital.

The study included patients of either sex diagnosed with diabetes mellitus of any duration, established as per American Diabetes Association (ADA) guidelines (random blood sugar >200 mg/dL or fasting blood sugar >126 mg/dL). They were further classified into diabetes mellitus type 1 (previous history of diabetic ketoacidosis) and type 2 (those with no history of ketoacidosis). Participants were interviewed for collection of information regarding demographics (age, sex), lifestyle characteristics (diet, exercise, smoking, alcohol consumption). Documentation from latest laboratory investigation reports documented in clinical records was used to derive biochemical parameters. At the time of recruitment anthropometric measurements including weight, height, body mass index (BMI; kg/m^2^) and waist circumference were carried out. Blood pressure of the participants was measured at the time of recruitment in the sitting position in the right arm to the nearest 2 mmHg with a mercury sphygmomanometer.

The patients were categorized into three age groups (<45years, 45-65years and >65years). The patients were either on oral medications, on insulin or on both. Compliance of the patients was assessed using blood sugar level in fasting or random and HbA1c according to American Diabetic Association criteria, and patient self-description regarding his diet, lifestyle and adherence to prescribed medication dose. On the basis of BMI, the patients were either under-weight (<18.5kg/m^2^), normal (18.5-24.9kg/m^2^), over-weight (25-30kg/m^2^) or obese (>30kg/m^2^).

The patients were assessed for the presence or absence of any other comorbid condition like neuropathy, nephropathy, retinopathy and dyslipidemia. Neuropathy was assessed using pinprick sensations, ankle reflexes and vibration perception threshold using Neurothesiometer. Inability to perceive the sensation at any one site was considered abnormal. In addition, ankle reflexes were also assessed with a percussion hammer. The diagnosis of diabetic retinopathy was made by an ophthalmologic examination that included fundoscopy or retinal photography and measurement of visual acuity. The diagnosis of nephropathy was confirmed from laboratory parameters including micro-albuminuria from clinical records.

Statistical software (SPSS v. 21) was used for data input and analysis. Cross tables were made between presence or absence of foot ulcer and compared with the involvement of a particular risk factor in it. Chi-square test for independence was used to test the significance of association between discrete variables. The dependent variable includes the presence or absence of diabetic foot ulcer. Findings with a *P* value less than 0.05 were considered significant.

## RESULTS

Demographics of the patients are shown in Table-I. Out of 2052 patients, 807 were male and 1247 were females. Total 1255 were above 45 years of age. There were 356 patients with diabetic foot ulcer. Patients with normal blood pressure were 308 in number while 948 were hypertensive. Another 1473 patients were overweight and 32 were in obese category. Patients having poor compliance with the medications, diet and lifestyle modifications were 1530 in number. Total 1996 patients were with co-morbid conditions, 1774 patients developed neuropathy, 324 retinopathies, 57 nephropathies, and 1547 had dyslipidemia.

**Table-I:** Comparison of Demographics of study population.

Parameters		Patients with DFU (n=356)	Patients without DFU (n=1696)	Total (n=2052)	P value
Gender	Male	147(41.3%)	660(38.9%)	807(39.3%)	0.219
	Female	209(58.7%)	1036(61.1%)	1245(60.7%)	
Age	<45	131(38.8%)	666(39.3%)	797(38.8%)	0.598
	45 – 65	204(57.3%)	922(54.4%)	1126(54.9%)	
	>65	21(5.9%)	108(6.4%)	129(6.3%)	
Medication	Oral	106(29.8%)	1139(67.2%)	1245(60.7%)	0.000
	Insulin	2(0.5%)	16(0.9%)	18(0.9%)	
	Combination	248(69.7%)	541(31.9%)	789(38.5%)	
Type of Diabetes Mellitus	DM1	131(36.8%)	666(39.3%)	797(38.8%)	0.209
	DM2	225(63.2%)	1030(60.7%)	1255(61.2%)	
Compliance	Good	105(29.5%)	417(24.6%)	522(25.4%)	0.032
	Poor	251(70.5%)	1279(75.4%)	1530(74.6%)	
Waist	>80 for Females & >90 for Males	294(82.6%)	356(21%)	650 (31.7%)	0.000
	<80 for Females & <90 for Males	62(17.4%)	1340(79%)	1402(68.3%)	
BMI	Underweight	1(0.3%)	22(1.3%)	23(1.1%)	0.003
	Normal	241(67.7%)	1232(72.6%)	524(25.5)	
	Overweight	105(29.5%)	419 (24.7%)	1473(71.8%)	
	Obese	9(2.5%)	23(1.4%)	32(1.6%)	
Blood Pressure	Low	2(0.6%)	1(0.06%)	3(0.1%)	0.118
	Normal	50(14.1%)	258(15.2%)	308(15%)	
	Pre-Hypertension	144(40.4%)	649(38.3%)	793(38.6%)	
	Hypertension	160(44.9%)	788(46.46%)	948(46.2%)	
Comorbidity	Yes	356(100%)	1640(96.7%)	1996(97.3%)	0.000
	No	0(0.0%)	56(3.3%)	56(2.7%)	
Neuropathy	Yes	349(98.1%)	1425(84.1%)	1774(86.5%)	0.000
	No	7(1.9%)	271(15.9%)	278(13.5%)	
Nephropathy	Yes	13(3.6%)	44(2.6%)	57(2.8%)	0.000
	No	343(96.4%)	1652(97.4%)	1995(97.2%)	
Retinopathy	Yes	100(28.1%)	223(13.1%)	323(15.7%)	0.000
	No	256(71.9%)	1473(86.9%)	1729(84.3%)	
Dyslipidemia	Yes	220(61.8%)	1327(78.2%)	1547(75.4%)	0.000
	No	136(38.2%)	369(21.8%)	505(24.6 %)	

The correlation of diabetic foot ulcer with different conditions are shown in Table I and II. Development of diabetic foot ulcer was strongly associated with use of combination therapy, poor compliance, higher waist, obesity and co-morbid conditions like, neuropathy and dyslipidemia. Correlation of different factors in patients without foot ulcer is shown in Table-III.

**Table-II:** Significant predictors of DFU.

Predictors	Patients with DFU (n=356)	
Neuropathy	Yes	349(98.1%)
	No	7(1.9%)
Waist	<80 for Females & <90 for Males	294(82.6%)
	>80 for Females & >90 for Males	62(17.4%)
Compliance	Good	105(29.5%)
	Poor	251(70.5%)
Medication	Oral	106(29.8%)
	Insulin	2(0.5%)
	Combination	248(69.7%)
BMI	Underweight	1(0.3%)
	Normal	241(67.7%)
	Overweight	105(29.5%)
	Obese	9(2.5%)
Dyslipidemia	Yes	220(61.8%)
	No	136(38.2%)
Nephropathy	Yes	13(3.6%)
	No	343(96.4%)
Retinopathy	Yes	100(28.1%)
	No	256(71.9%)

**Table-III:** Non-Significant predictors of DFU.

Parameters	Patients with DFU (n=356)	
Gender	Male	147(41.3%)
	Female	209(58.7%)
Age	<45	131(38.8%)
	45 – 65	204(57.3%)
	>65	21(5.9%)
Type of Diabetes Mellitus	DM1	131(36.8%)
	DM2	225(63.2%)
Blood Pressure	Low	2(0.6%)
	Normal	50(14.1%)
	Pre-Hypertension	144(40.4%)
	Hypertension	160(44.9%)

## DISCUSSION

In literature, the risk factors of foot ulceration varied in studies, and some of them were similar. We found that patients taking a combination of insulin and oral medication were more likely to develop foot ulcer than were patients whose diabetes was managed with either oral glycemic agent or insulin alone. This result was statistically significant (p value 0.00). Reason may be because of the fact that with time, diabetes progressed and became less responsive to treatment.^8^ Moreover, use of many medications may decrease patient compliance to therapy,^9^ which led to loss of glycemic control^10^ and resultant increase in DFU risk.^11^ An additional explanation could be that when patients started insulin, they may already had diabetes for a longer duration, with greater associated complications already present. It could also be because insulin had played the role of confounder. The reasons might be the unequal number of patients in the two groups (those developing foot ulcer were 356 as opposed to 1696 who did not develop foot ulcer). This finding was compatible with other studies like Yazdanpanah et al.^12^ a prospective cohort study done in Iran and Mohammed et al.^6^ a cross sectional study done in Iraqi patients. Sample size was low in both these studies as compared to our study. In a systematic review, seven studies out of 16 reported an association between DFU and insulin treatment.^13^ Further studies may be needed to elaborate on this variable by eliminating the possible confounding factors and providing more details.

Another well-known risk factor identified was distal neuropathy (98.1% people with DFU had peripheral neuropathy). This was congruous with many studies including Yazdanpanah L et al.^12^ and Says et al.^7^ It was indeed worrying as patients with distal neuropathy could endure minor trauma without being aware of the injury until it worsens. It had been suggested that this condition can be prevented with an improved health education program offering advice on protection in the home and at work, good hygiene and physical examination (using mirrors to examine feet may help).

Comorbidities were a strong risk factors for DFU as also shown by Says et al.^7^ This was probably because comorbidities result in increased number of medication which can cause poor compliance. It resulted in poor glycemic control and hence increased risk of DFU. On the other hand, Mohammed et al.^6^ contradicted with this findings. He emphasized that an increase in demand for glycemic control, increased patient’s compliance with medical advice.^14^ This improved glycemic control and fewer complications from hyperglycemic attacks.^10^,^15^

Greater BMI and increased waist circumference were a risk factor for DFU.^16^ Both these results were found statistically significant. In literature, the results were statistically insignificant, which was contradicting. The possible reason could be due to the presence of higher foot pressure in those with higher body mass index (BMI) might decrease intensively the normal blood circulation pattern at the lower extremities leading to DFU.

Poor compliance and dyslipidemia were found statistically significant risk factors for DFU. However, some studies did not found them associated with diabetic foot ulcer.^6^ Another finding of this study was predominance of male patients developing DFU. However, from p value this difference was insignificant. Moreover, this result was in accordance with Yazdanpanah L et al.^12^ which reported similar finding probably because of more foot exposure to risk factors due to outdoor activity and plantar pressure in males. But again, their finding was significant in univariate analysis and not in multivariate analysis.

A statistically insignificant finding of the present study pointed out that nephropathy was not an independent risk factor for DFU. This is inconsistent with Says et al^7^ and American Diabetes Association consensus group. It was not possible to explain this variation and may be a feature only present in our patients. More work may be required with higher number of patients to confirm our findings.

This study was similar in calculation of gender based calculation of foot ulcer with other local studies. While Younis et al.^17^ have documented in local study that females were more commonly presenting with foot ulcer, we have also similar findings. Their findings of association with neuropathy, peripheral arterial disease, female sex, increasing age, duration of diabetes and high HbA1c had some similarities and some dissimilarities with our study. Khan A et al.^18^ also documented similar findings in the local population, with female dominance. The possible reason could be that the tertiary care centers are mostly working in the morning, which is a convenient time for this gender to visit.

The occurrence of DFUs mostly in middle aged subjects had been reported by several researchers. In the present study, we found that people with age between 45 and 65 years had highest percentage of DFUs (57.3%) and thus support the findings of previous workers like Mohamed et al.^6^ and Says et al.^7^ and other studies.^19^ However, the result was not found statistically significant. Retinopathy was found to be a risk factor for diabetic foot ulcer and this result was also statistically significant. This result is in consistence with other studies.^6^

Hypertension and type 2 DM^16^,^20^ were also independent risk factors for development of diabetic foot ulcer, but results were statistically insignificant. Hypertension was usually associated with type 2 diabetes, which resulted in higher risk of cardiovascular diseases and mortality. Such association leads to the development of nephropathy, retinopathy, and diabetic cardiomyopathy. Since systemic arterial hypertension increased the risk of micro and macro-vascular injuries, the risk of Peripheral Arterial Disease also increased.^21^ Type 2 DM had associated complications for foot ulcer. It included mechanical changes in the conformation of the bony architecture of the foot, peripheral neuropathy, and atherosclerotic peripheral arterial disease. As a result, the patient may have less tissue epithelization, consumption of oxygen, nutrient transportation, and cell detoxification resulting in ulceration in the extremities.

### Limitations of the study

First, our patients were from a teaching hospital, and this may had resulted in selection bias. However, our hospital was the referral center and the focal point of diabetes in the area. Hence, variation could be a strong point. We did not consider some potential confounders in the occurrence of new foot ulceration such as health care provision level and patient behavioral factors like training on their foot care and residency (rural or urban area). Factors like previous history of DFU, foot deformity, amputation, diabetes duration, educational level, marital status, job activity, smoking, alcohol, tobacco chewing, glycemic control (HbA1c), and decreased peripheral pulses were not analyzed. This study was cross-sectional rather than longitudinal design that can affect the reliability of study conclusions. Therefore, in order to confirm the results of this study, a longitudinal large-scale study should be performed. Our data was not collected using a questionnaire. Therefore, we could not avail the advantages of a questionnaire in which patient had enough time to fill in the required data. Finally, differences in methods of neuropathy assessment may also had affected the results.

The strength of this study was its low cost. We had missing data too. The results of this study could support the suggestion to reduce DFU incidence. However, we still need further studies with a larger sample size and longer follow-up period to support these findings.

## CONCLUSION

The association of DFU was not documented in the catchment area of our area population before. Some factors like combination of oral and injectable medication in management, higher waist circumference, greater BMI, neuropathy, dyslipidemia, retinopathy and poor compliance were strongly linked. However, middle age, male gender, Type 2 diabetes and Hypertension were insignificantly co-related. These finding provided support for a multifactorial etiology of DFU. We need further studies in larger population to support these findings.

### Authors’ Contribution

**MIHK:** Conceived, reviewed, editing of manuscript, final approval of manuscript, submission of manuscript.

**UA:** Material & Methodology, Data entry, Designed tables & graphs.

**FZ:** Introduction & discussion writing.

**ZAK:** Statistical analysis, Designed tables & graphs.
